# The Dynamic Comprehensive Evaluation of the Importance of Cutting Parameters in the Side Milling TC4 Process Using an Integrated End Mill

**DOI:** 10.3390/ma17112744

**Published:** 2024-06-04

**Authors:** Xingfu Zhao, Yanzhong Wang, Lin Jin, Zemin Zhao, Daxun Yue, Yuyuan Wang, Zengcheng Wang, Zongxu Dai

**Affiliations:** 1 School of Mechanical Engineering & Automation, Beihang University, Beijing 100083, China; yzwang63@126.com; 2 AECC Harbin Dongan Engine Co., Ltd., Harbin 150066, China; 1367460680@163.com (L.J.); zeminzhao666@163.com (Z.Z.); 3 School of Mechatronics Engineering, Harbin Institute of Technology, Harbin 150001, China; 22B908080@stu.hit.edu.cn (D.Y.); 23S108304@stu.hit.edu.cn (Y.W.); 23S108308@stu.hit.edu.cn (Z.W.); 23S108300@stu.hit.edu.cn (Z.D.)

**Keywords:** dynamic comprehensive evaluation, cutting parameters, comprehensive importance, radar map

## Abstract

In the cutting process, there are many parameters that affect the cutting effect, and the same parameter has different degrees of influence on different performance indicators, which makes it difficult to select key parameters for parameter optimization and parameter combination evaluation while considering multiple performance indicators at the same time. The process of titanium alloy milling with an integrated end mill is studied herein. The values of cutting tool flank face wear and material removal rates are obtained with experimental and analytical methods. Numerical characteristics and causes of the cutting tool flank face wear at different stages are also analyzed. The dynamic, comprehensive evaluation method based on the double incentives model is used to evaluate the dynamic, comprehensive importance of cutting parameters in view of the problem of considering multiple performance indicators and the characteristics of the dynamic change in performance indicators in the cutting process. According to the result of a dynamic, comprehensive evaluation, the cutting parameters with the highest comprehensive importance are selected. Finally, the radar map is used to plot the comprehensive importance of the cutting parameters. The overall comprehensive importance of each cutting parameter is intuitively displayed as well. As a result of the research, the dynamic, comprehensive evaluation method based on the double incentives model has a good application value in the evaluation of tool performance in the cutting process and can quickly select the best tool performance parameter combination; it is established that the most comprehensive parameter is the cutting speed, and the cutting width is the second most important. In turn, the comprehensive importance of the cutting depth is the lowest.

## 1. Introduction

As is well known, metal cutting is a common way to manufacture metal parts [1]. In the cutting process, the same parameters (tool geometric parameters and tool operating parameters) have different degrees of influence on different performance indicators. To obtain the best cutting performance, those parameters are adjusted depending on their influence degree [2]. However, in this process, the tool geometric parameters and tool operating parameters total at least ten parameters, which increases the difficulty of the performance adjustment. In addition, different performance indicators have different units and optimization directions; in order to be able to achieve high-performance machining, multiple performance indicators are often selected at the same time to carry out cutting research, resulting in difficulties in selecting a clear standard to measure each performance indicator. Therefore, when studying the cutting process based on multiple objectives, it is important to explore the degree of influence of the parameters on multiple performance indicators at the same time, select key parameters, and improve the research process.

The parameter sensitivity refers to the degree to which the parameter affects the performance indicator. Today, a number of scholars often conduct range analyses to obtain the parameter sensitivity. The main principle of a range analysis is that the greater the range value *R*, the greater the degree of influence. The other researchers use modern evaluation methods to calculate the importance of each parameter. Feng et al. [3] took a micro-texture cutter milling GH4169 as the research object; used a micro-texture size, a micro-crater diameter, and a micro-crater spacing as the research parameters; set up an L_16_(3^4^) orthogonal test; obtained the cutting force values corresponding to different parameter combinations using DEFORM 11.0 software; and calculated the cutting force range values of the three parameters using the range analysis method. The results show that the pit-spacing range is the largest and the pit-texture size range is the smallest, indicating that the pit-spacing sensitivity is the highest and the pit-texture size sensitivity is the smallest in terms of cutting force. Kong et al. [4] took the laser-assisted turning titanium alloy TC6 as the research object, established the FEM model of the laser-assisted turning process with DEFORM software, established the L_9_(3^3^) orthogonal test to obtain tool wear values corresponding to different turning parameter groups, and analyzed the sensitivity of cutting parameters to tool wear with the range analysis method. The results show that the sensitivity of cutting speed is the highest and the sensitivity of cutting depth is the lowest. Li et al. [5] established a FEM model of the milling nickel-based superalloy process using ABAQUS. Taking milling force and milling temperature as performance indicators, the range analysis of the cutting parameters was carried out according to milling force and milling temperature, respectively, using the range analysis method. The analysis results show that for milling force, feed per tooth > cutting depth > spindle speed; for milling temperature feed per tooth > cutting depth ≈ spindle speed. Yang et al. [6] conducted experiments on milling titanium alloy with a micro-textured ball-end milling cutter. The diameter, depth, spacing, and distance from the cutting edge of a single pit were studied as the parameters, and the surface residual stress of titanium alloy was taken as the performance indicator to analyze the influence of the same parameters on the surface residual stress of the workpiece. The results show that pit spacing > distance from the pit to the cutting edge > pit diameter > pit depth. Li et al. [7] studied the influence of parameters on performance indicators during the optimization of the process parameters such as tool type, feed speed, and cutting depth in Ti6Al4V dry turning. Considering the nonlinear relationship between various targets, grey correlation analysis (GRA) was used to convert each indicator into the corresponding grey correlation coefficient. Then, the kernel principal component analysis (KPCA) was used to extract the kernel principal component and determine the corresponding weights to represent the relative importance of each target.

In the process of researching the sensitivity and comprehensive importance of the parameters, it is found that the sensitivity of parameters refers to the degree of influence of parameters on a performance indicator. Commonly used methods include the range analysis method and response surface methodology (RSM). However, when two or more performance indicators need to be considered at the same time, due to the different effects of the same parameter on different performance indicators, it is easy to find that the same parameter has a strong impact on performance indicator 1 and a low impact on performance indicator 2. Therefore, the concept of parameter sensitivity cannot clearly measure the importance of the same parameter to multiple performance indicators at the same time, especially contradictory performance indicators such as tool wear and material removal rate, when the cutting parameters increase, tool wear and material removal rates will increase together; it is difficult to find the balance point between the two performance indicators. Among them, “simultaneously” considering multiple performance indicators means that the values of multiple performance indicators are input into the comprehensive evaluation method to obtain the comprehensive evaluation values of the evaluated object, and the mapping relationship is that multiple performance indicators correspond to a set of comprehensive evaluation results. Although the above scholars also studied the problem of multiple performance indicators, the parameter sensitivity analysis was carried out separately according to the number of performance indicators, and multiple performance indicators were not considered at the same time in the analysis of parameter sensitivity. Therefore, the concept of the comprehensive importance of parameters gradually emerged. The comprehensive importance of parameters is a qualitative concept, which is mainly used to describe the importance of the same parameter to two or more performance indicators “at the same time”. Each parameter itself is taken as the evaluation object and the value of the performance indicator is taken as the evaluation basis. The comprehensive importance of parameters to multiple performance indicators can be obtained using comprehensive evaluation methods, which include the grey correlation method, the fuzzy comprehensive evaluation method, and the grey–fuzzy analytic hierarchy process. Yue et al. [8] used DEFORM finite element simulation software to establish the FEM model of the milling process of titanium alloy with a milling cutter to obtain the tool wear rate value and used analytical methods to obtain the material removal rate value. They evaluated the comprehensive importance of tool parameters and cutting parameters through the grey–fuzzy analytic hierarchy process method and selected the four parameters with the highest comprehensive importance. The result shows that the most important parameters are the clearance angle, helix angle, feed per tooth, and cutting depth. However, the grey–fuzzy analytic hierarchy process is a static comprehensive evaluation method, which evaluates the comprehensive importance of parameters according to the performance indicator value of a certain stage, but the cutting process is a dynamic change in the performance indicator value, such as tool wear gradually increasing with the increase in cutting distance. Therefore, it is of great significance to consider the variation in performance indicator values in multiple stages while conducting a comprehensive importance evaluation.

When milling difficult materials such as titanium alloys, the high strength and hardness of those materials at high temperatures accelerate the rate of tool wear, resulting in rapid tool failure and reduced machining efficiency [9,10,11]. In this paper, the key problems of large tool wear and low machining efficiency in the milling process of titanium alloy are investigated. Taking the side milling of titanium alloy with an end mill as the research object, the phenomenon existing in the cutting process is studied and the causes are analyzed. Considering the dynamic change in performance indicators, the tool wear and material removal rate are selected as the evaluation basis, and the comprehensive evaluation model is composed of the grey–fuzzy analytic hierarchy process and the dynamic comprehensive evaluation method based on the double incentives model. The model is used to evaluate milling parameters comprehensively, and the dynamic comprehensive evaluation values of each milling parameter are obtained. Finally, the radar map of the comprehensive importance of cutting parameters is plotted to visually show the comprehensive importance of each cutting parameter.

## 2. Dry Milling of Titanium Alloy with Integrated End Mill

### 2.1. Tool, Equipment and Performance Indicators of the Milling Process

**Figure 1:**
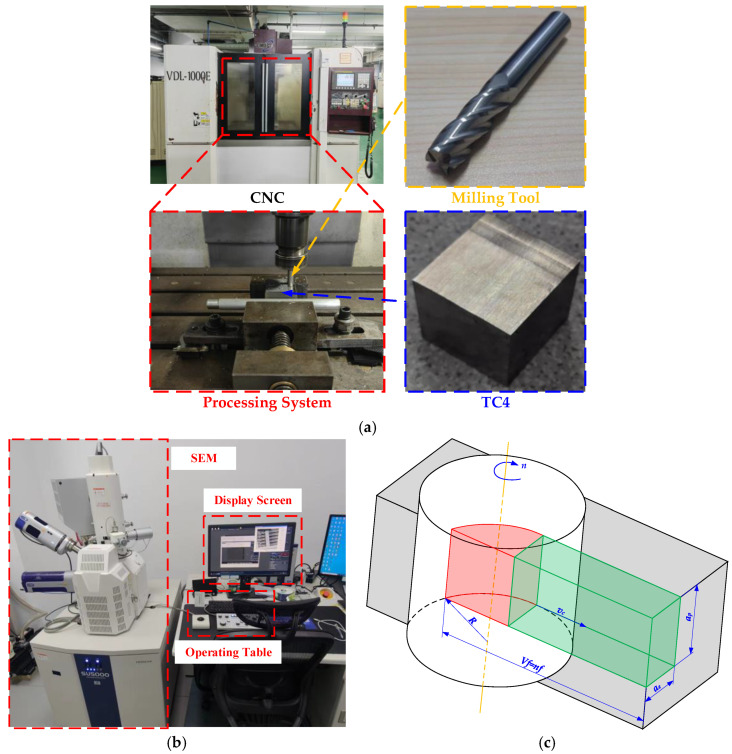
Tool, Workpiece, Equipment, Performance Indicator, and Measuring Instrument of the Milling Process: (**a**) Test Processing Equipment; (**b**) Scanning Electron Microscope; (**c**) Material Removal Rate Diagram.

Tool life is an important criterion for evaluating tool performance. Tool flank face wear is one of the important indicators to evaluate the tool life [12]. According to the literature [13], the dry milling of titanium alloy with an uncoated carbide end mill is studied in this paper. The end mill edge flank face wear and material removal rate are taken as performance indicators. The values of the tool wear and material removal rates under different cutting parameters are obtained using experimental and analytical methods. The cutter is an integrated carbide end mill (four edges, 10 mm diameter, 75 mm length); the material, in turn, is carbide YG6. The workpiece is a cube (length × width × height = 50 mm × 50 mm × 50 mm) made of TC4. The CNC machine tool is a three-axis CNC milling machine from the Shenyang Machine Tool Factory (VDL-1000E, Shenyang Machine Tool Factory, Shenyang, China). The tool flank face wear is quantified through the wear width produced with the tool flank face, which is represented by *VB* [14]. Scanning electron microscopy (SU5000, HITACHI, Tokyo, Japan) is used to measure the tool flank face wear of the end mill. Figure 1 shows the tool, workpiece, equipment, performance indicator, and measuring instrument used for the milling test. Equation (1) is the calculation equation of the material removal rate.

(1) $$V_{\mathrm{Material}\;\mathrm{removal}\;\mathrm{rate}}=\left(\begin{array}{l} \frac{1}{2}\cdot R^{2}\cdot \arccos\left(1-\frac{a_{e}}{R}\right)-\frac{1}{2}\cdot \left(R-a_{e}\right)\cdot \sqrt{2\cdot R\cdot a_{e}-{a_{e}}^{2}}+\\ \left(\frac{50\cdot v_{c}\cdot f_{z}\cdot z}{6\cdot \pi \cdot R}-\sqrt{2\cdot R\cdot a_{e}-{a_{e}}^{2}}\right)\cdot a_{e} \end{array}\right)\cdot a_{p}$$

where *V*_Material removal rate_ is the volume of material removal per unit time, in mm^3^/s; *R* is the tool radius, in mm; *a_e_* is the cutting width, in mm; *v_c_* is the cutting speed, in m/min; *f_z_* is the feed per tooth, in mm/z; *z* is the number of tool teeth; and *a_p_* is the cutting depth, in mm.

### 2.2. Table of the Cutting Test Parameters

An orthogonal test is an efficient research method to reduce the number of tests, to achieve the purpose of predicting the best results through a small number of tests, and to achieve a reduction in test cost and time [15,16,17]. In this paper, the cutting speed, feed per tooth, cutting depth, and cutting width are selected for orthogonal experiments. SPSS 25 software is used to establish the orthogonal test table without considering the interaction between parameters [18,19]. Table 1 is the orthogonal test table of the milling parameters.

### 2.3. Cutting Test Results and Analysis

**Figure 2:**
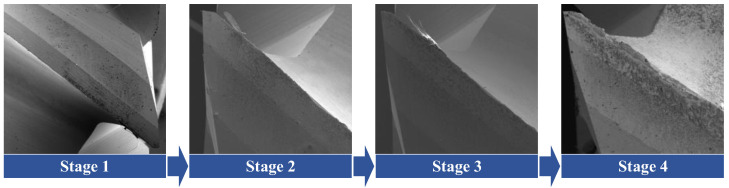
Tool Wear Changes at Different Stages.

According to the size of the titanium alloy workpiece, the cutting distance of each stage is selected by cutting 30 times along the side length of the workpiece. That is, the cutting distance of each stage is 1500 mm; the four cutting stages are represented by *t*_1_, *t*_2_, *t*_3_, and *t*_4_; and the cutting distance represented in each cutting stage is 1500 mm, 3000 mm, 4500 mm, and 6000 mm. After that, the tool face wear value of the integral end mill is measured using a scanning electron microscope, and the tool face wear values in four stages are obtained. Since the titanium alloy workpiece is rectangular and the cutting mode is repeated, the material removal rate of the same group of cutting parameters at different stages is the same. Table 2 shows the performance indicator data of the flank face wear and material removal rates. Figure 2 shows the variation in tool wear at different stages of the tool. With the increase in the cutting distance, the tool wear degree gradually increases. The first stage of tool wear is not obvious. When the second stage is reached, tool wear occurs. The degree of tool wear in the third stage has little change from that in the second stage. When the final stage is reached, the tool wear is obvious.

**Figure 3:**
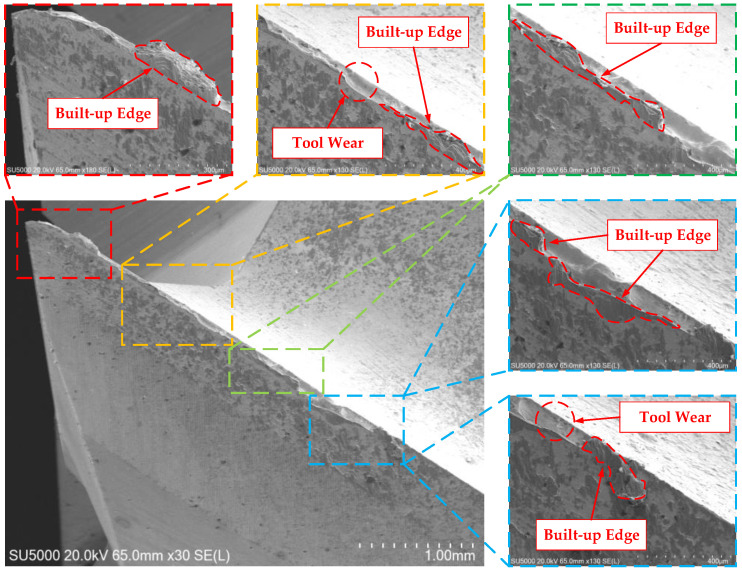
Built-Up Edge on the Cutting Edge of the End Mill.

According to the tool wear curve from the literature [20], the tool wear gradually increases with the increase in the cutting distance. However, the tool wear data in Table 2 demonstrate that the wear of some parameter combinations in the front and back stages has problems of decline, resulting in little change in the measured tool wear; the reason for the decline is the built-up edge. Under certain pressure and temperature conditions, the chips bond to the cutting edge and form a built-up edge [21]. Therefore, the tool wear can be measured visually. The presence of a built-up edge has a great influence on the measurement of the tool wear, which causes a deviation in the measurement results and leads to the phenomenon of a decrease in the tool wear. The reason why the tool wear does not change much is that the built-up edge replaces the cutting edge during the cutting process, which is equivalent to the protective film on the outer layer of the cutting edge. The built-up edge reduces the rate of the tool wear so that the tool wear does not change much in the front and back stages. The reason for the sharp increase in tool wear is that the built-up edge falls off. Built-up edges are characterized by the forming process, falling off, forming again, and falling off again [22]. When a built-up edge replaces the cutting edge for cutting, the built-up edge falls off under the impact of the chip, and then the cutting edge replaces the built-up edge for cutting, resulting in a sharp increase in tool wear. Figure 3 shows the built-up edge produced during the titanium alloy milling using the integrated end mill, which is bonded to part of the cutting edge.

**Figure 4:**
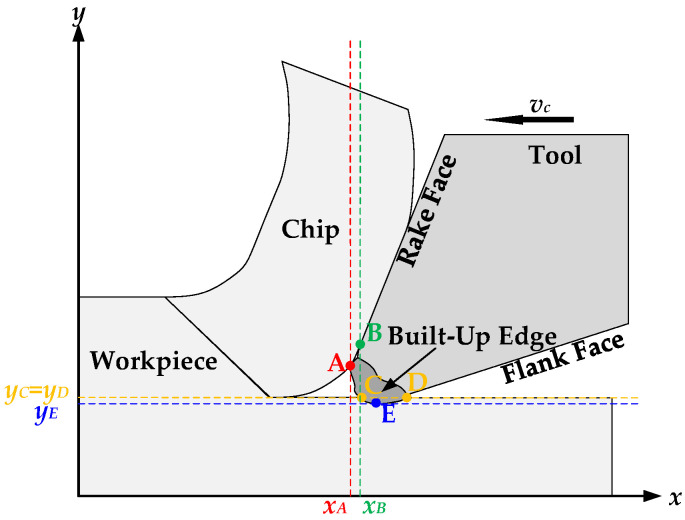
Schematic Diagram of Cutting Workpiece with Built-Up Edge Instead of Cutting Edges.

Figure 4 shows the schematic diagram of the workpiece cutting with a built-up edge instead of cutting edges. It can be observed from the figure that point A on the built-up edge comes into contact with the workpiece earlier than point B on the cutting edge (*x*_A_ < *x*_B_), and the built-up edge replaces the cutting edge for cutting at a certain time during the cutting process.

The formation of a built-up edge is random; therefore, it can appear that the depth of point E on the built-up edge is greater than that of point C and point D on the processed surface of the workpiece. That leads to the conclusion that the actual cutting width is greater than the theoretical cutting width, reducing the surface quality of the workpiece (*y*_E_ < *y*_C_ = *y*_D_).

## 3. Comprehensive Evaluation of Cutting Parameter Importance Using Dynamic Comprehensive Evaluation Method Based on Double Incentives Model

### 3.1. Dynamic Comprehensive Evaluation Method Based on Double Incentives Model

The forms of motivation include explicit incentive and implicit incentive. “Explicit incentive” is a kind of incentive aimed at the development status of the evaluated object, which only considers the status of the evaluation value at different stages and lacks the analysis of the change in the evaluation value at different moments in time. “Implicit incentive” is a kind of incentive that can analyze the evaluation value of different development trends of the evaluated object [23].

In order to make the evaluation method more comprehensive and reasonable, it is not only necessary to analyze the state of the evaluated object but also important to analyze the development trend of the evaluated object. Therefore, the dual incentive model is used. This model is an efficient combination of the explicit incentive model and the implicit incentive model [24]. According to different incentive conditions of “explicit incentive” and “implicit incentive” models, nine types of double incentive models can be considered, as in Table 3.

**Figure 5:**
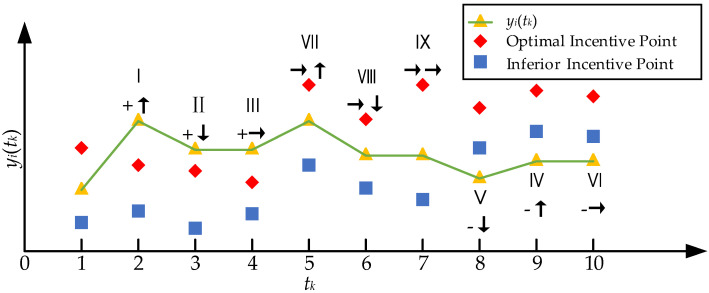
Schematic Diagram of Double Incentives Model.

In Table 3, “+” indicates that an explicit incentive is an optimal incentive, “-” indicates that an explicit incentive is an inferior incentive, and “→” indicates that an explicit incentive is a non-incentive. “↑” indicates that an implicit incentive is an upward incentive, “↓” indicates that an implicit incentive is a downward incentive, and “→” indicates that an implicit incentive is a non-incentive. The scheme of double incentive types is shown below in Figure 5. Since *t*_1_ is the initial point of the diagram, there is only an explicit incentive and no implicit incentive. *t*_2_~*t*_10_ are the nine types of double incentives shown in Table 3.

**Figure 6:**
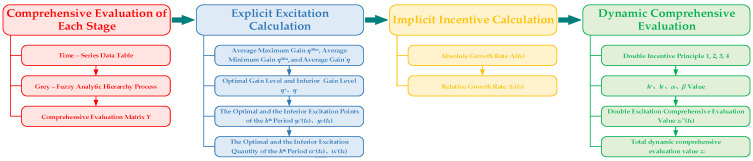
Dynamic Comprehensive Evaluation Method Based on Double Incentives Model.

Figure 6 shows the calculation flow of the dynamic comprehensive evaluation method based on the double incentives model. The application of the method includes four steps. These steps are the comprehensive evaluation process of each stage, the explicit incentive calculation process, the implicit incentive calculation process, and the double incentives calculation process.

The comprehensive evaluation matrix *Y* is obtained through the comprehensive evaluation process of each stage. The optimal and inferior incentive values of *υ_i_^+^*(*t_k_*), *υ_i_^−^*(*t_k_*) at each stage are obtained through the explicit incentive model. The absolute growth rate ∆*_i_*(*t_k_*) and the relative growth rate ∆^′^*_i_*(*t_k_*) are obtained using the implicit incentive model. The total dynamic comprehensive evaluation value *z_i_* of the evaluated object is obtained using the double incentives model.

According to the literature [25], as shown in the sequential stereoscopic data table (Table 4), *n* parameters are set for evaluation. *s_i_* represents the *i*th parameter, and there are *m* micro-evaluation bases for performance indicators; *x_ij_*(*t_k_*) is the *j*th performance indicator value corresponding to the *i*th parameter in the *t^k^* period.

In this paper, the grey–fuzzy analytic hierarchy process is used to process the performance indicator data in the temporal stereoscopic data table to obtain a comprehensive evaluation matrix *Y*. This method is composed of a fuzzy comprehensive evaluation method, a grey correlation method, and an analytic hierarchy process, and has the function of the dimensionless processing of performance indicators [26,27,28,29]. It can solve the problems existing in the cutting process such as fuzziness [30], grey data quantity [31], confusion in multi-objective evaluation systems, and the non-unity of indicator units.

According to the literature [32,33], Equation (2) is obtained. This equation represents the comprehensive evaluation numerical matrix *B_k_* of *n* evaluation objects at the *kth* stage. Then, the evaluation matrix *B_k_* of *T* stages is transformed and combined to obtain the comprehensive evaluation matrix *Y*. The expression form of the comprehensive evaluation matrix *Y* is shown below (Equation (3)).

(2) $$B_{k}=\left[\begin{array}{cccc} y_{1}\left(t_{k}\right) & y_{2}\left(t_{k}\right) & \ldots & y_{n}\left(t_{k}\right) \end{array}\right]$$

(3) $$Y=\left[\begin{array}{cccc} y_{1}\left(t_{1}\right) & y_{1}\left(t_{2}\right) & \cdots & y_{1}\left(t_{T}\right)\\ y_{2}\left(t_{1}\right) & y_{2}\left(t_{2}\right) & \cdots & y_{2}\left(t_{T}\right)\\ \vdots & \vdots & \vdots & \vdots \\ y_{n}\left(t_{1}\right) & y_{n}\left(t_{2}\right) & \cdots & y_{n}\left(t_{T}\right) \end{array}\right]$$

### 3.2. The Calculation Flow of the Double Incentives Dynamic Evaluation Method

According to Section 3.1, the comprehensive evaluation matrix *Y* is obtained, and the explicit incentive model, implicit incentive model, and double incentives model are used to calculate the total dynamic comprehensive evaluation value *z_i_* of the evaluated object.

- According to the explicit model in Section 3.1, mathematically, it can be written using Equations (4)–(7) [34]. According to these equations, the comprehensive evaluation matrix *Y* is processed, and *υ_i_^+^*(*t_k_*), *υ_i_*^−^(*t_k_*) are obtained.

(4) $$\left\{\begin{array}{l} \eta^{\max}=\underset{i}{\max}\left(\frac{1}{T-1}\overset{T-1}{\underset{k=1}{\Sigma }}\left(y_{i}\left(t_{k+1}\right)-y_{i}\left(t_{k}\right)\right)\right)\\ \eta^{\min}=\underset{i}{\min}\left(\frac{1}{T-1}\overset{T-1}{\underset{k=1}{\Sigma }}\left(y_{i}\left(t_{k+1}\right)-y_{i}\left(t_{k}\right)\right)\right)\\ \bar{\eta }=\frac{1}{n\left(T-1\right)}\overset{n}{\underset{i=1}{\Sigma }}\overset{T-1}{\underset{k=1}{\Sigma }}\left(y_{i}\left(t_{k+1}\right)-y_{i}\left(t_{k}\right)\right) \end{array}\right.$$

(5) $$\left\{\begin{array}{l} \eta^{+}=\bar{\eta }+k^{+}\left(\eta^{\max}-\bar{\eta }\right)\\ \eta^{-}=\bar{\eta }-k^{-}\left(\bar{\eta }-\eta^{\min}\right) \end{array}\right.$$

(6) $$\left\{\begin{array}{l} \eta^{+}=y_{i}^{+}\left(t_{k}\right)-y_{i}\left(t_{k-1}\right),(k=2,3,\ldots ,T)\\ \eta^{-}=y_{i}^{-}\left(t_{k}\right)-y_{i}\left(t_{k-1}\right),(k=2,3,\ldots ,T) \end{array}\right.$$

(7) $$\left\{\begin{array}{l} \upsilon_{i}^{+}\left(t_{k}\right)=y_{i}\left(t_{k}\right)-y_{i}^{+}\left(t_{k}\right),y_{i}\left(t_{k}\right)>y_{i}^{+}\left(t_{k}\right)\\ \upsilon_{i}^{-}\left(t_{k}\right)=y_{i}^{-}\left(t_{k}\right)-y_{i}\left(t_{k}\right),y_{i}^{-}\left(t_{k}\right)>y_{i}\left(t_{k}\right) \end{array}\right.$$

where *η^max^*, *η^min^*, and *η* are the average maximum gain, average minimum gain, and average gain. *η^+^* and *η^+^* are optimal and inferior gain levels; *k^+^* and *k^−^* are the corresponding floating coefficients, *k^+^* and *k^−^*∈(0,1]; *y_i_^+^*(*t_k_*) and *y_i_*^−^(*t_k_*) are the optimal and inferior incentive points, which can be obtained by substituting the optimal and inferior gain levels into Equation (7); and *υ_i_^+^*(*t_k_*) and *υ_i_*^−^(*t_k_*) are the optimal and inferior incentive quantities obtained by the *i*th evaluated object at the *t_k_* stage, respectively.

- According to the literature [24], the evaluation value of “implicit incentive” is determined by Definition 1 and Equation (8). Comprehensive evaluation matrix *Y* is calculated below through this equation. In addition to this, the absolute growth rate ∆*_i_*(*t_k_*) and relative growth rate ∆^′^*_i_*(*t_k_*) required by the double incentives model are obtained.

**Definition** **1.** *Let ∆_i_(t_k_) be the absolute growth rate of the ith evaluation object at the kth stage and ∆^′^_i_(t_k_) be the relative growth rate of the ith evaluation object at the kth stage. Then, the “implicit incentive” evaluation value z_i_* *^⊙^(t_k_) of the evaluated object at the t_k−1_ ~ t_k_ stage is obtained according to Equation (8).*

(8) $$\left\{\begin{array}{l} {z_{i}}^{⊙}\left(t_{k}\right)=y_{i}(t_{k})\left(1+\frac{\alpha }{1+e^{-\Delta_{i}(t_{k})}}+\frac{\beta }{1+e^{-{\Delta^{\prime }}_{i}(t_{k})}}\right)\\ \Delta_{i}(t_{k})=\frac{\left(y_{i}(t_{k})-y_{i}(t_{k-1})\right)}{t_{k}-t_{k-1}}\\ {\Delta^{\prime }}_{i}(t_{k})=\frac{\Delta_{i}(t_{k})}{\left(\frac{1}{n-1}\sum_{j=1,j\neq i}^{n}\Delta_{j}(t_{k})\right)} \end{array}\right.$$

*where α and β are undetermined parameters (α + β ≥ 1), the purpose of which is to make z_i_* *^⊙^(t_k_) non-negative. Otherwise, the “negative performance” will not occur. α/(1 + e^−^* *^∆i(tk)^), in turn, is the incentive coefficient of the absolute growth rate, which represents the incentive degree of the absolute growth trend (which can rise or fall) of the evaluated object s_i_ in the* *t_k−1_ ~ t_k_ stage. β/(1 + e^−∆′i(tk)^) is the incentive coefficient of the relative growth rate, indicating the incentive degree of the evaluated object s_i_ at the stage of t_k−1_ ~ t_k_ relative growth trend (which can rise or fall), that is, compared with other evaluated objects, s_j_ (j = 1, 2, …, n; j ≠ i) is the degree of motivation obtained by comparison. In addition, it needs to set the initial growth rate ∆_i_(t_k_) = ∆^′^_i_(t_k_) = 0.*

- According to Definition 2 in the literature [24], the numerical calculation of the double incentives model is obtained using Equation (9). The dynamic comprehensive evaluation value z_i_^**^(*t_k_*) of the *i*th evaluated object at the *k*th stage is calculated through this equation. The *υ_i_^+^*(*t_k_*) and *υ_i_^−^*(*t_k_*) are obtained from the explicit incentive model and the absolute growth rate ∆*_i_*(*t_k_*). The relative growth rate ∆^′^*_i_*(*t_k_*), in turn, is obtained from the implicit incentive model. The undetermined parameters *α* and *β* and the optimal and inferior incentive factors *h^+^* and *h^−^* are determined using Equations (10)–(13).

**Definition** **2.** The “explicit incentive” evaluation value z_i_^⊕^(t_k_) and the “implicit incentive” evaluation value z_i_^⊙^(t_k_) are arithmetically fused, and the integrated evaluation value z_i_^**^(t_k_) after fusion is the double incentives evaluation value of the ith evaluated object at the kth stage. This is shown in Equation (9).

(9) $${z_{i}}^{**}\left(t_{k}\right)=y_{i}\left(t_{k}\right)+y_{i}\left(t_{k}\right)\left(\frac{\alpha }{1+e^{-\Delta_{i}\left(t_{k}\right)}}+\frac{\beta }{1+e^{-{\Delta^{\prime }}_{i}(t_{k})}}\right)+h^{+}\upsilon_{i}^{+}\left(t_{k}\right)-h^{-}\upsilon_{i}^{-}\left(t_{k}\right)$$

*where h^+^ and h^−^ (h^+^, h^−^ > 0) are optimal and inferior incentive factors, respectively.*

**Double Incentives Rule 1. Double Incentives Quantity Total Proportion Rule.** In general, for *n* evaluation objects, the total amount of “explicit incentive” and the total amount of “implicit incentive” are required to be proportional to each other, and the ratio between those is expressed by the parameter *ξ,* whose equation is expressed using Equation (10).

(10) $$\xi =\frac{h^{+}\sum_{i=1}^{n}\sum_{k=1}^{T}\upsilon_{i}^{+}\left(t_{k}\right)+h^{-}\sum_{i=1}^{n}\sum_{k=1}^{T}\upsilon_{i}^{-}\left(t_{k}\right)}{\sum_{i=1}^{n}\sum_{k=1}^{T}y_{i}\left(t_{k}\right)\left(\frac{\alpha }{1+e^{-\Delta_{i}\left(t_{k}\right)}}+\frac{\beta }{1+e^{-{\Delta^{\prime }}_{i}\left(t_{k}\right)}}\right)}$$

where the parameter *ξ* can be determined according to the preference of the decision-maker. If the decision-maker prefers “explicit incentive”, then *ξ* > 1; if the decision-maker prefers “implicit incentives” *ξ* < 1, the general range of the parameter *ξ* is [0.2, 5].

**Double Incentives Rule 2. The Principle of Double Incentives Moderation.** The sum of the “explicit incentive” factor and the “implicit incentive” factor is required to be 1, and its expression is shown in Equation (11).

(11) $$h^{+}+h^{-}+\sum_{k=2}^{T}\left(\frac{\alpha }{1+e^{-\Delta_{i}\left(t_{k}\right)}}+\frac{\beta }{1+e^{-{\Delta^{\prime }}_{i}\left(t_{k}\right)}}\right)=1$$

**Double Incentives Rule 3. Absolute Incentive and Relative Incentive Total Proportion Characteristic Principle**. The total absolute incentive is obtained with the evaluated object and it is required to be proportional to the relative incentive total. The equation of the ratio *r* between them is shown in Equation (12).

(12) $$r=\frac{\sum_{i=1}^{n}\sum_{k=1}^{T}y_{i}\left(t_{k}\right)\frac{\alpha }{1+e^{-\Delta_{i}\left(t_{k}\right)}}}{\sum_{i=1}^{n}\sum_{k=1}^{T}y_{i}\left(t_{k}\right)\frac{\beta }{1+e^{-{\Delta^{\prime }}_{i}\left(t_{k}\right)}}}$$

where parameter *r* can be determined according to the preference of the decision-maker, and *r* > 1 can be set if “absolute incentive” is selected; if one decides to select the “relative incentive” then *r* < 1; the general range of parameter *r* is [0.2, 5].

**Double Incentives Rule 4. Principle of Conservation of Total Quantity of Optimal and Inferior Incentive.** For *n* evaluation objects, it is required that the total amount of optimal incentive and the total amount of inferior incentive be equal. This is shown below in Equation (13).

(13) $$h^{+}\sum_{i=1}^{n}\sum_{k=1}^{T}\upsilon_{i}^{+}\left(t_{k}\right)=h^{-}\sum_{i=1}^{n}\sum_{k=1}^{T}\upsilon_{i}^{-}\left(t_{k}\right)$$

The values of *h^+^*, *h^−^*, *α*, and *β* can be calculated using Equations (10)–(13), and a specific form of Equation (9) is determined. Finally, by synthesizing all stages of {*t_k_*}, the total dynamic comprehensive evaluation value *z_i_* of the *i*th evaluated object at every moment of time *T* is obtained. This is shown below in Equation (14).

(14) $$z_{i}=\overset{T}{\underset{k=1}{\Sigma }}\tau_{k}z_{i}\left(t_{k}\right)$$

where *τ_k_* is the time factor. Usually, series of increasing {*τ_k_*} are used. Also, this parameter can be set as *τ_k_* = 1 if there are no specific requirements.

### 3.3. Comprehensive Evaluation of the Cutting Parameters Importance Based on the Dynamic Evaluation Method of the Double Incentives Model

- Comprehensive Evaluation of Each Stage

First of all, the grey–fuzzy analytic hierarchy process is used to conduct a comprehensive evaluation of the performance indicator data in Table 2 at each stage. The comprehensive importance values of four cutting parameters at all four stages are obtained. The evaluation results are shown below. The comprehensive evaluation matrix *Y* is obtained through the combination of *B*_1_, *B*_2_, *B*_3_, and *B*_4_.

$$\left\{\begin{array}{l} B_{1}={\left[\begin{array}{cccc} 0.9160 & 0.7675 & 0.7294 & 0.9127 \end{array}\right]}^{\tau }\\ B_{2}={\left[\begin{array}{cccc} 0.8980 & 0.7688 & 0.7954 & 0.9127 \end{array}\right]}^{\tau }\\ B_{3}={\left[\begin{array}{cccc} 0.8080 & 0.8935 & 0.7714 & 0.8407 \end{array}\right]}^{\tau }\\ B_{4}={\left[\begin{array}{cccc} 0.9760 & 0.7015 & 0.6574 & 0.9127 \end{array}\right]}^{\tau } \end{array}\right.$$

$$Y=\left[\begin{array}{cccc} 0.9160 & 0.8980 & 0.8080 & 0.9760\\ 0.7675 & 0.7688 & 0.8395 & 0.7015\\ 0.7294 & 0.7954 & 0.7714 & 0.6574\\ 0.9127 & 0.9127 & 0.8407 & 0.9127 \end{array}\right]$$

- Calculation of the Optimal and Inferior Incentive Quantities using the Dominant Excitation Model

According to Equations (4)–(6), the floating coefficients *k^+^* and *k*^−^ in Equation (5) are set to 0.3 [32], and the average maximum gain *η^max^* = 0.02, average minimum gain *η^min^* = −0.024, and average gain *η* = −0.0065 are calculated. The optimal gain level *η^+^* = 0.00145, the inferior gain level *η^−^* = −0.01175, and the values of the optimal and inferior incentive points of the cutting parameters at different stages are shown in Table 5.

According to the values of optimal and inferior incentive points in Table 5, the optimal and inferior incentive quantities of cutting parameters at different stages are obtained using Equation (7), as shown in Table 6.

- The absolute growth rate ∆*_i_*(*t_k_*) and relative growth rate ∆^′^*_i_*(*t_k_*) of the implicit incentive model

Since each cutting distance is set to be the same during the cutting process, *l_k_*_+1_ − *l_k_* = 1, the absolute growth rate ∆*_i_*(*t_k_*) and relative growth rate ∆^′^*_i_*(*t_k_*) of the implicit incentive model are calculated according to Equation (8). The calculation results are shown in Table 7.

- Determination of undetermined constants *h^+^*, *h*^−^, *α*, and *β*

According to Equations (10)–(13), substituting *ξ* = *r* = 1, the values of *h^+^*, *h*^−^, *α*, and *β* are shown in Table 8.

- Calculation of the total dynamic comprehensive evaluation value *z_i_* of each cutting parameter

According to Equations (8) and (9), the implicit incentive evaluation value *z_i_*^⊙^(*t_k_*) and the cutting parameter evaluation value z_i_^**^(*t_k_*) at each stage are obtained. According to Equation (14), *τ_k_* = 1, the total dynamic comprehensive evaluation value *z_i_* of each parameter is obtained. The calculation results are shown in Table 9 and Table 10.

**Figure 7:**
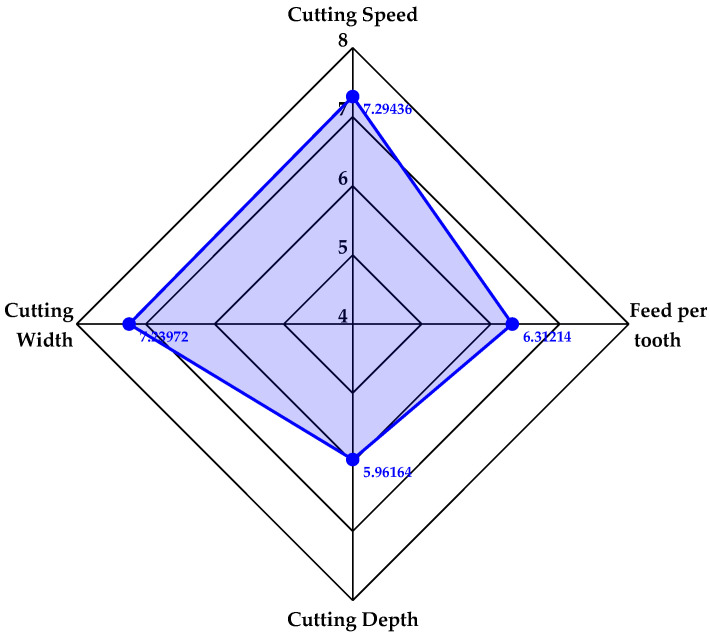
Radar Map for Dynamic Evaluation of Comprehensive Importance of Cutting Parameters.

Comprehensive evaluation refers to a method that adopts systematic and standardized evaluation methods to assess multi-index systems. Radar map analysis is a typical intuitive graphic data analysis method in comprehensive evaluation [35,36]. According to the results of the dynamic evaluation in Table 10, the radar map for the dynamic comprehensive evaluation of the comprehensive importance of cutting parameters is plotted in Figure 7. Figure 7 demonstrates that cutting speed is the parameter with the highest comprehensive importance, cutting width ranks second, and the difference between the evaluation values of the two parameters is small. Feed per tooth ranks third and cutting depth has the lowest comprehensive importance. Therefore, the cutting speed and cutting width can be chosen to be studied emphatically in the later research.

### 3.4. Comparative Analysis of Evaluation Results

Using the range analysis method, the influence degree of parameters of different performance indicators is obtained, and the comprehensive evaluation results are compared. The range analysis is performed using the tool wear and material removal rate values of the last stage. Table 11 shows the range analysis table for tool wear and Table 12 shows the range analysis table for the material removal rate.

It can be seen from Table 11 that the cutting width is the parameter that has the greatest influence on tool wear, the cutting speed ranks second, and the cutting depth has the least influence. And, it can be seen from Table 12 that the cutting width is the parameter that has the greatest influence on the material removal rate, the feed per tooth ranks second, and the cutting speed has the least influence. The range analysis shows that the cutting width is the most important parameter to the tool wear and material removal rates, which is consistent with the comprehensive evaluation results. Although the cutting speed has the least effect on the material removal rate, it can rank second in the comprehensive evaluation. This is because there is little difference in the range of the cutting speed, feed per tooth, and cutting depth, and the weight value of the tool wear is higher than the weight value of the material removal rate in the comprehensive evaluation process. The effects of feed rate per tooth and cutting depth on tool wear and material removal rate are similar to the comprehensive evaluation results. Therefore, the results of the comprehensive evaluation and the range analysis have high similarity, and the results of the comprehensive evaluation are reliable.

## 4. Conclusions

In the cutting process, the performance indicators are different, the optimization direction is different, and the parameters have different effects on the performance indicator; there is no clear standard to measure each performance indicator, and the data of the performance indicators in the cutting process will change with the increase in the cutting distance. In this paper, titanium alloy milling with an end milling cutter is studied, and the characteristics and causes of the tool wear value change at different stages of each parameter combination in the test process are analyzed. The dynamic comprehensive evaluation method based on the double incentives model is used to assess the comprehensive importance of each cutting parameter. As a result of the evaluation, the cutting parameter with the highest comprehensive importance is selected. And, the reliability of the comprehensive evaluation results is verified using the comparison between the range analysis method and the comprehensive evaluation results. The following conclusions can be made as a result of the conducted research:

- In the process of milling titanium alloy with an end mill, it is found that it has a serious built-up edge phenomenon. The tool wear of two adjacent stages has decreased and slightly changed. The reason for these problems is that the built-up edge bond on the cutting edge affects the measurement of the tool wear;
- There is a phenomenon of the sharp increase in the tool wear of two adjacent stages, which is caused by the built-up edge instead of the edge cutting and falling off at a certain time, caused by the cutting edge continuing to cut;
- According to the dynamic comprehensive evaluation results, cutting speed > cutting width > feed per tooth > cutting depth. The comprehensive importance of cutting speed is the highest, cutting width ranks second, the difference between the evaluation values of the two parameters is small, the feed per tooth ranks third, and the comprehensive importance of cutting depth is the lowest;
- Through the range analysis, it is found that the range analysis results are similar to the comprehensive evaluation results. Therefore, the result of a comprehensive evaluation is reliable.

The dynamic, evaluation method based on the double incentives model is an evaluation method with high universality and applicability. The method can be applied in other fields, such as mechanical manufacturing, mechanical design, urban management, economics, etc. However, certain prerequisites are required for the method to be used to evaluate the evaluated object:

- The research object is a dynamic research object;
- The number of performance indicators based on the comprehensive evaluation should be at least two, and the performance indicator itself is a quantifiable indicator;
- Among the performance indicators involved in the evaluation, at least one performance indicator must be sufficient to change over time.

## Figures and Tables

**Table 1:** Milling Parameters for the Orthogonal Test.

No.	Cutting Speed (m/min)	Feed Per Tooth (mm/z)	Cutting Depth (mm)	Cutting Width (mm)
1	50	0.10	2.0	0.2
2	50	0.15	3.0	0.4
3	50	0.20	4.0	0.6
4	62.5	0.10	3.0	0.6
5	62.5	0.15	4.0	0.2
6	62.5	0.20	2.0	0.4
7	78.125	0.10	4.0	0.4
8	78.125	0.15	2.0	0.6
9	78.125	0.20	3.0	0.2

**Table 2:** Performance Indicators.

No.	Tool Flank Face Wear *VB* (µm)				Material Removal Rate *V* (mm^3^/s)
	*t*_1_	*t*_2_	*t*_3_	*t*_4_	
1	8.04	22.84	23.82	28.80	4.0590
2	7.38	40.68	18.12	32.40	18.3277
3	8.37	67.37	67.97	129.00	49.0778
4	8.40	42.50	43.44	204.00	22.4844
5	11.80	56.69	59.92	67.70	15.5452
6	10.80	50.62	71.94	93.80	20.7068
7	9.19	47.16	49.12	85.20	25.4980
8	9.84	55.95	61.22	70.00	28.9156
9	8.92	64.61	70.29	75.50	19.6166

**Table 3:** Double Incentives Model Types.

Model Type	Ⅰ	Ⅱ	Ⅲ	Ⅳ	Ⅴ	Ⅵ	Ⅶ	Ⅷ	Ⅸ
Explicit Incentive	+	+	+	-	-	-	→	→	→
Implicit Incentive	↑	↓	→	↑	↓	→	↑	↓	→
Model Type	+ ↑	+ ↓	+ →	- ↑	- ↓	- →	→ ↑	→ ↓	→ →
Double Incentives	Ⅰ	Ⅱ	Ⅲ	Ⅳ	Ⅴ	Ⅵ	Ⅶ	Ⅷ	Ⅸ

**Table 4:** Temporal Stereoscopic Data Table.

	*t*_1_				*t*_2_				…	*t*_T_			
	*x*_1_	*x*_2_	…	*x_m_*	*x*_1_	*x*_2_	…	*x_m_*	…	*x*_1_	*x*_2_	…	*x_m_*
*s*_1_	*x*_11_(*t*_1_)	*x*_12_(*t*_1_)	…	*x*_1*m*_(*t*_1_)	*x*_11_(*t*_2_)	*x*_12_(*t*_2_)	…	*x*_1*m*_(*t*_2_)	…	*x*_11_(*t*_T_)	*x*_12_(*t*_T_)	…	*x*_1*m*_(*t*_T_)
*s*_2_	*x*_21_(*t*_1_)	*x*_22_(*t*_1_)	…	*x*_2*m*_(*t*_1_)	*x*_21_(*t*_2_)	*x*_22_(*t*_2_)	…	*x*_2*m*_(*t*_2_)	…	*x*_21_(*t*_T_)	*x*_22_(*t*_T_)	…	*x*_2*m*_(*t*_T_)
…	…	…	…	…	…	…	…	…	…	…	…	…	…
*s_n_*	*x_n_*_1_(*t*_1_)	*x_n_*_2_(*t*_1_)	…	*x_nm_*(*t*_1_)	*x_n_*_1_(*t*_2_)	*x_n_*_2_(*t*_2_)	…	*x_nm_*(*t*_2_)	…	*x_n_*_1_(*t*_T_)	*x_n_*_2_(*t*_T_)	…	*x_nm_*(*t*_T_)

**Table 5:** Values of Optimal and Inferior Incentive Points of Cutting Parameters at Different Stages.

	*t*_2_		*t*_3_		*t*_4_	
	*y_i_^+^*(*t*_2_)	*y_i_*^−^(*t*_2_)	*y_i_^+^*(*t*_3_)	*y_i_*^−^(*t*_3_)	*y_i_^+^*(*t*_4_)	*y_i_*^−^(*t*_4_)
Cutting Speed	0.91745	0.90425	0.89945	0.88625	0.80945	0.79625
Feed Per Tooth	0.76895	0.75575	0.77025	0.75705	0.89495	0.88175
Cutting Depth	0.73085	0.71765	0.79685	0.78365	0.77285	0.75965
Cutting Width	0.91415	0.90095	0.91415	0.90095	0.84215	0.82895

**Table 6:** Incentive Quantity of Cutting Parameters in Different Stages.

	*t*_1_		*t*_2_		*t*_3_		*t*_4_	
	*υ_i_^+^*(*t_k_*)	*υ_i_*^−^(*t_k_*)	*υ_i_^+^*(*t_k_*)	*υ_i_*^−^(*t_k_*)	*υ_i_^+^*(*t_k_*)	*υ_i_*^−^(*t_k_*)	*υ_i_^+^*(*t_k_*)	*υ_i_*^−^(*t_k_*)
Cutting Speed	0	0	0	0.00625	0	0.07825	0.16655	0
Feed Per Tooth	0	0	0	0	0.12325	0	0	0.18025
Cutting Depth	0	0	0.06455	0	0	0.01225	0	0.10225
Cutting Width	0	0	0	0	0	0.06025	0.07055	0

**Table 7:** Absolute Growth Rate ∆*_i_*(*t_k_*) and Relative Growth Rate ∆^′^*_i_*(*t_k_*) of the Implicit Incentive Model.

	*t*_1_	*t*_2_	*t*_3_	*t*_4_
∆_Cutting Speed_	0	−0.018	−0.09	0.168
∆_Feed Per Tooth_	0	0.0013	0.1247	−0.192
∆_Cutting Depth_	0	0.066	−0.024	−0.114
∆_Cutting Width_	0	0	−0.072	0.072
∆^′^_Cutting Speed_	0	−0.802377415	−9.407665505	−2.153846154
∆^′^_Feed Per Tooth_	0	0.08125	−2.011290323	−4.571428571
∆^′^_Cutting Depth_	0	−11.85628743	1.930294906	−7.125

**Table 8:** Table of Undetermined Constants.

*h* ^+^	*h* ^−^	*α*	*β*
0.021436005	0.03700759	0.334738076	0.323618222

**Table 9:** Implicit Incentive Evaluation Value *z_i_*^⊙^(*t_k_*).

	*t*_1_	*t*_2_	*t*_3_	*t*_4_
*z* _Cutting Speed_^⊙^(*t_k_*)	0.942767167	0.917824274	0.816273157	0.991092835
*z* _Feed Per Tooth_^⊙^(*t_k_*)	0.78992773	0.791848676	0.907575428	0.708564863
*z* _Cutting Depth_^⊙^(*t_k_*)	0.750714379	0.804206534	0.804498813	0.664064389
*z* _Cutting Width_^⊙^(*t_k_*)	0.939370735	0.939370735	0.849386382	0.928674321

**Table 10:** Dynamic Comprehensive Evaluation of Comprehensive Importance of Parameters.

	*z*_i_^⊙^(*t*_1_)	*z*_i_^⊙^(*t*_2_)	*z*_i_^⊙^(*t*_3_)	*z*_i_^⊙^(*t*_4_)	*z*
Cutting Speed	1.858767167	1.813801660	1.598950031	2.022843461	7.294362319
Feed Per Tooth	1.557427730	1.560648676	1.842331896	1.351732679	6.312140981
Cutting Depth	1.480114379	1.621213877	1.571934489	1.288374426	5.961637171
Cutting Width	1.852070735	1.852070735	1.670588385	1.864990092	7.239719946

**Table 11:** Tool Wear Range Analysis Table.

	Cutting Speed	Feed Per Tooth	Cutting Depth	Cutting Width
*K*_1_	190.20	318.00	192.60	172.00
*K*_2_	346.45	170.10	311.90	211.40
*K*_3_	230.70	298.30	281.90	403.00
*k*_1_	63.40	106.00	64.20	57.33
*k*_2_	115.48	56.70	103.97	70.47
*k*_3_	76.90	99.43	93.97	134.33
*R*	52.08	49.30	39.77	77.00
	Cutting Width > Cutting Speed > Feed Per Tooth > Cutting Depth			

**Table 12:** Material Removal Rate Range Analysis Table.

	Cutting Speed	Feed Per Tooth	Cutting Depth	Cutting Width
*K*_1_	71.46	52.04	53.68	39.22
*K*_2_	58.74	62.79	60.43	64.53
*K*_3_	74.03	89.40	90.12	100.48
*k*_1_	23.82	17.35	17.89	13.07
*k*_2_	19.57	20.93	20.14	21.51
*k*_3_	24.68	29.80	30.04	33.49
*R*	5.11	12.45	12.15	20.42
	Cutting Width > Feed Per Tooth > Cutting Depth > Cutting Speed			

## Data Availability

The raw data supporting the conclusions of this article will be made available by the authors on request.
